# The challenge of patient recruitment into early phase clinical trials: Is time-effective academic translational research feasible? Lessons from a phase I/II trial in patients with B-cell deficiencies

**DOI:** 10.3389/fmed.2026.1844041

**Published:** 2026-07-29

**Authors:** Maddalena Marconato, Simon Jäger, Christian M. Tegeler, Christopher Hackenbruch, Helmut R. Salih, Juliane S. Walz, Birgit Federmann, Jonas S. Heitmann

**Affiliations:** 1 Department of Medical Oncology and Hematology, University Hospital Zurich, Zurich, Switzerland; 2 Clinical Collaboration Unit Translational Immunology, Department of Internal Medicine, University Hospital, Tübingen, Germany; 3 Department of Peptide-based Immunotherapy, Institute of Immunology, University and University Hospital Tübingen, Tübingen, Germany; 4 Dr. Margarete Fischer-Bosch Institute of Clinical Pharmacology, Stuttgart, Germany; 5 Department of Clinical Pharmacology, University of Tübingen, Tübingen, Germany; 6 Institute of Clinical Pharmacology, Paracelsus Medical University, Nürnberg, Germany; 7 Department of Women’s Health, University Hospital Tübingen, Tübingen, Germany; 8 Cluster of Excellence iFIT (EXC2180) “Image-Guided and Functionally Instructed Tumor Therapies”, University of Tübingen, Tübingen, Germany; 9 German Cancer Consortium (DKTK), partner site Tübingen, a partnership between DKFZ and University Hospital Tübingen, Tübingen, Germany

**Keywords:** early-phase clinical trials, eligibility criteria, investigator-initiated trials, patient recruitment challenges, translational research

## Abstract

Recruiting patients in early-phase clinical trials poses significant challenges, particularly when targeting specific patient populations. This emerged during the phase I/II B-pVAC-SARS-CoV-2 trial investigating CoVac-1, a peptide-based T-cell activator developed to provide T-cell-mediated protection against severe COVID-19 in individuals with inherited or acquired B-cell deficiencies who were unable to mount adequate antibody responses following approved vaccination AGAINST SARS-CoV-2. Despite promising phase I results in healthy volunteers and high patient interest, recruitment of immunocompromised subjects proved substantially more difficult than initially anticipated. Strict inclusion and exclusion criteria, combined with the complexity of patient profiles, required assessing 1,024 candidates at one site to finally recruit 61 subjects and extended the recruitment process to over 8 months. Our experience highlights how rigid eligibility criteria and lengthy protocol adaptation processes may unintentionally limit access to promising investigational treatments for patients with urgent unmet medical needs. More flexible, patient-tailored approaches, whenever safety is not compromised, together with more pragmatic approaches to protocol adaptation, for instance driven by expedited consultations with the DSMB, may help improve recruitment, while preserving patient safety and trial integrity, particularly in academic early-phase clinical research.

## Introduction

Recruiting patients for early-phase clinical trials is often marked by significant challenges, particularly when these trials are investigator-initiated (IIT), academic-based and targeting specific patient sub-populations with complex medical needs (1–3). Previous reports already highlighted obstacles such as stringent inclusion and exclusion criteria, limited patient pools, ethical considerations and logistical constraints that can significantly delay trial-specific milestones and limit results’ generalizability (2, 4, 5). These challenges are amplified in trials focusing on rare conditions or vulnerable groups, where identifying and enrolling eligible participants becomes a major bottleneck. Despite advances in clinical trial methodologies, flexible recruitment strategies remain an underdeveloped area, posing a persistent hurdle in translating experimental treatments into practice.

Focusing on our IIT, we tried to further define potential obstacles to time-effective recruitment. The Phase I/II, multicenter B-pVAC-SARS-CoV-2 (Phase I/II multi-center safety and immunogenicity trial of multi-peptide vaccination (CoVac-1) to prevent COVID-19 infection in adults with B-cell/antibody deficiency, NCT04954469) trial (6, 7) investigating a novel peptide-based T-cell activator against SARS-CoV-2 (8) in patients with B-cell immunodeficiencies, was developed with the aim to provide an effective immunization against the new coronavirus to patients with the inability to mount humoral immune responses (6, 7). While the trial’s results are detailed elsewhere (6, 7), this work focuses on the recruitment challenges encountered at a trial site in Germany. Through this lens, we aim to highlight the need for innovative and adaptable recruitment strategies, shedding light on an often-overlooked aspect of clinical research that is crucial for the successful translation of novel therapies into clinical practice, which may not always be fully addressed by increasing the number of participating trial sites alone.

## Methods

### Overview of trial execution

The B-pVAC-SARS-CoV-2 trial took place between July 2021 and April 2023 and entailed three sections. During Phase I Part I, 14 patients were included and received one dose of CoVac-1. Based on positive preliminary results, Phase II was initiated, and 40 patients were recruited to receive a single administration of CoVac-1. Finally, the safety and efficacy of two subsequent doses of CoVac-1 were tested during a novel Phase I Part II, with the enrollment of 14 new patients. Phase I Part I and Phase I Part II were conducted at the Clinical Collaboration Unit (CCU) Translational Immunology, University Hospital Tübingen, exclusively, while Phase II was extended to two further trial sites, namely the Institute of Clinical Cancer Research, Krankenhaus Nordwest, University Cancer Center, Frankfurt; and the Department of Hematology, Oncology, and Cancer Immunology, Campus Benjamin Franklin, Charité-Universitätsmedizin Berlin.

CoVac-1 is a peptide-based T-cell activator with reported good tolerability and efficacy in inducing specific T-cell response against SARS-CoV-2 epitopes (8). Eligibility was based on the presence of an inherited or acquired B-cell deficiency, defined as either one among (i) hypogammaglobulinemia (IgG below a given threshold), (ii) clinical need for IgG-replacement therapy, or (iii) ongoing therapy with a B-cell depleting agent (i.e., Rituximab or Obinutuzumab). Previous history of proven COVID-19, as well as the presence of antibodies against SARS-CoV-2 represented major exclusion criteria (inclusion and exclusion criteria are outlined in detail in Table 1). Of note, at the time of protocol development, the immunogenicity endpoint relied on the assessment of induced SARS-CoV-2-specific T-cell responses in previously unexposed individuals. Prior infection with SARS-CoV-2 (possibly documented by specific antibodies) could have confounded interpretation of novel induced immune responses and therefore represented exclusion criteria. Given the rapidly evolving pandemic situation and limited knowledge regarding the duration and quality of natural immunity available at the time, a conservative approach was adopted. Furthermore, we specify that, since the study was initiated during the ongoing COVID-19 pandemic, several eligibility criteria were defined based on the best available evidence at the time. A deeper evaluation of possible obstacles to recruitment was not possible, as the intention of the team was to timely deliver a new tool to patients at high risk of severe infections while the virus was actively spreading.

**Table 1:** Detailed inclusion and exclusion criteria for trial participants.

Inclusion criteria	Exclusion criteria
Adult (≥18 years) male or non-pregnant, non-lactating female Primary antibody deficiency syndrome or Secondary antibody deficiency syndrome, defined by one of the following: IgG <5.5 g/l^1^ Ongoing substitution of immunoglobline for hypogammaglobinemia Anti-CD20 antibody (monospecific) therapy for malignant disease: after combined Anti-CD20 antibody therapy with chemotherapy (e.g., fludarabin, cyclophosphamid, bendamustin, anthracycline, vincristin) (within 1–6 months post therapy) ongoing or up to 6 months after single agent Anti-CD20 antibody therapy ongoing or up to 6 months after combined Anti-CD20 antibody therapy with BTK-inhibitors or BCL2-inhibitors^1^ Anti-CD20 antibody maintenance therapy Ability to understand and voluntarily sign an informed consent form Ability to adhere to the study visit schedule and other protocol requirements Female patients of childbearing potential (FCBP) and male patients with partners of childbearing potential, who are sexually active, must agree to the use of two effective forms (at least one highly effective method) of contraception. This should be started from the signing of the informed consent and continue until 3 months after vaccination. Furthermore, contraception must be carried on by patients receiving B-cell depleting therapies for the whole duration of the treatment. Postmenopausal or evidence of non-child-bearing status. For women of childbearing potential: negative urine or serum pregnancy test within 7 days prior to study treatment. Postmenopausal or evidence of nonchildbearing status is defined as: Amenorrhoea for 1 year or more following cessation of exogenous hormonal treatments Luteinizing hormone (LH) and Follicle stimulating hormone (FSH) levels in the postmenopausal range for women under 50	Pregnant or lactating females Participation in any clinical trial with intake of any nonregistered vaccine product Any concomitant disease affecting the effect of the therapeutic vaccine or interfering with the study primary endpoint: Active infection Psychiatric disorders Known systemic anaphylaxis Prior or current infection with SARS-CoV-2 as assessed by medical history and/or by throat/nose swab (PCR) or serologically documented immunization against SARSCoV-2 (after infection or vaccination) Persisting symptoms developed after SARS-CoV-2 vaccination with an approved vaccine product at study inclusion History of Guillain-Barré syndrome HIV infection, chronic or active hepatitis B or C History of relevant CNS pathology or current relevant CNS pathology (e.g., seizure, paresis, aphasia, cerebrovascular ischemia/haemorrhage, severe brain injuries, dementia, Parkinsons disease, cerebellar disease, organic brain syndrome, psychosis, coordination or movement disorder, excluding febrile seizures as child) Baseline laboratory CD4 + T cell count <100 μL The following pre-existing medical conditions: Chronic liver failure defined as Child-Pugh Score ≥B Chronic renal failure defined as GFR < 40 mL/min/1,73 m2 Serious pre-existing cardiovascular disease such as NYHA ≥III Sickle cell anemia Patients presenting with any clinical, laboratory or radiological signs of tumor-progression Patient receiving active treatment with Proteosome-Inhibitors (e.g., Bortezomib), Phosphoinositid-3-Kinase-Inhibors (e.g., Idelalisib) 13. Known hypersensitivity to any of the components included in the CoVac-1 vaccine Pre-existing auto-immune disease except for Hashimoto thyroiditis and mild (not requiring immunosuppressive treatment) psoriasis Intention of receiving one dose of an already approved vaccine against SARS-CoV-2 before day 56

IgG, Immunoglobulin G; CNS, Central Nervous System; NYHA, New York Heath Association.

Amendment of criteria: ^1^Changed after amendment number 1 (30.09.2021) prior to initiation of phase II: original cut off = 4 g/L; additionally, the exclusion criterion “concomitant treatment with BTK-inhibitors and BCL2-inhibitors” was eliminated in the same amendment.

A total of 68 adult patients were included and treated in the clinical trial. Overall, 61 patients were enrolled at the CCU Translational Immunology, University Hospital Tübingen. Seven additional patients were recruited either in Berlin or Frankfurt and had no contact with the main study center, nor additional information about further potential candidates in these centers was made available to us. The present article will focus exclusively on the recruitment process developed in Tübingen.

Notably, reimbursement of travel expenses of up to €500 per participant was provided.

### Participants identification and enrollment process

The enrollment process was characterized by a continuous proactive identification of suitable candidates at the University Hospital Tübingen. Moreover, physicians working in the proximity of the trial center and involved in the treatment of patients affected with inherited or acquired B-cell deficiencies were informed about the trial by the study team. Furthermore, information about the study was posted on the homepage of the trial center and was given press attention, leading to the referral from other centers of subjects suffering from B-cell deficiencies, as well as to the unsolicited spontaneous candidacy of additional patients.

To expedite the recruitment process, the study team evaluated each single unsolicited application received by e-mail (i.e., spontaneous candidacy for the trial sent by patients who came across the trial and were not referred by physicians), identifying major inclusion and exclusion criteria. Subjects who missed inclusion or violated exclusion criteria were informed about their non-eligibility, while potentially eligible candidates were further assessed through a written questionnaire and a telephone interview with a physician. Based on study criteria, patients were either excluded or invited for a screening visit, as defined per protocol, which led to the final inclusion or exclusion of the candidate.

Potential participants who were identified at the local hospital during routine visits were informed about the existence of the trial by their treating physician (i.e., solicited candidates). In case these subjects stated their interest in trial participation, a further assessment of eligibility was performed by an investigator (in person during a routine visit or over the phone, through a dedicated interview). Again, individuals with any ineligibility criteria were excluded from the list of possible candidates. Otherwise, patients were invited for a screening visit.

### Data collection

No identifying data from non-eligible patients who agreed to undertake the questionnaire and the interview with an investigator was collected or stored. For the purpose of understanding the possible difficulties of the recruitment phase, a code was attributed to each exclusion criterion and their recurrence was documented for solicited and unsolicited candidates who underwent the first contact. For each candidate, the main reason for exclusion was recorded. The presence of concomitant exclusion criteria was not documented.

This posed the basis for subsequent amendments to the trial protocol, aimed at improving the enrollment procedure.

Data from patients who underwent a regular screening visit were collected in the eCRF, as per protocol. Study participants were required to sign a written informed consent, which had been previously revised and approved by the Ethics Committee of the Medical Faculty of the Eberhard Karls University of Tübingen and the University of Tübingen.

## Results

### Enrollment process and overview of excluded patients

**Figure 1:**
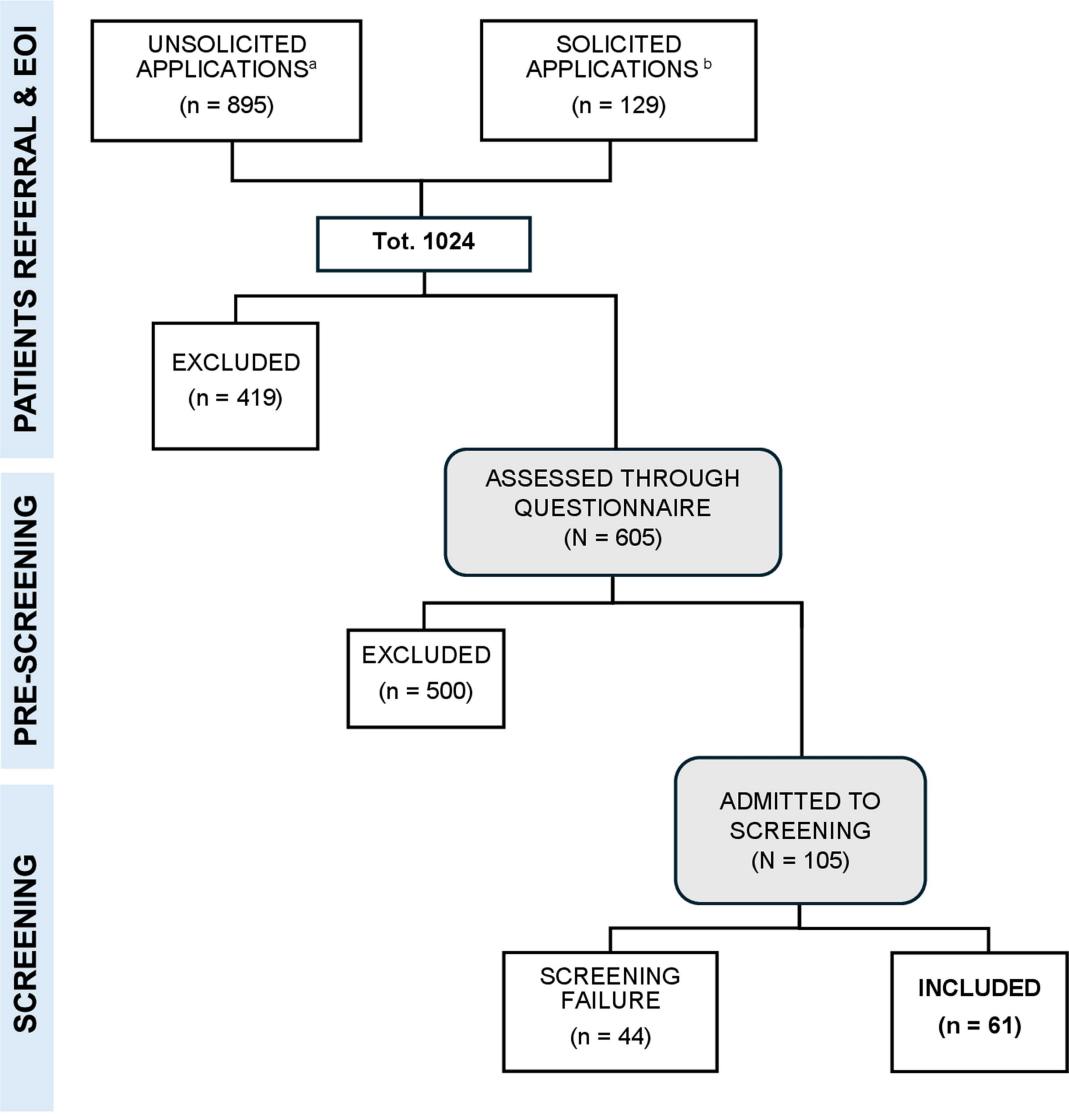
Overview of recruitment process. EOI, expression of interest. ^a^Patients who contacted the clinical trial team after being informed about the trial by their physicians or through the media. ^b^Patients identified at the University Hospital Tübingen and informed about the trial by the study team.

Overall, 1,024 subjects were scrutinized as potential trial participants, of which 895 were unsolicited candidates who contacted the clinical trial team and 129 were solicited candidates identified at the trial site by physicians who were informed about the trial populations but did not extensively assess patients for all trial-specific criteria (Figure 1). A total of 105 patients (10.2% of all candidates, 10.1% and 10.9% of unsolicited and solicited candidates, respectively) were admitted to the screening visit and 61 (5.9% of all candidates) were enrolled into the trial and treated.

Of note, 419 of the 895 patients that contacted the trial center at the University Hospital Tübingen were directly excluded, as they did not belong to the trial population. Most of these were affected with solid tumors and were interested in a new vaccine that could prevent severe COVID-19 in fragile individuals. The remaining 476 patients that expressed their interest reported an underlying disease or immune-system disorder that was consistent with the inclusion criteria. These subjects were further assessed for eligibility. After this second step, 385 patients resulted non-eligible, whereas 91 subjects were admitted to the screening visit.

Similarly, 115 subjects identified at the trial site as possible candidates were excluded based on the exclusion criteria.

Finally, after the required physical and laboratory assessment performed during the screening visit, 44 screening failures occurred.

### Most frequent reasons for exclusion of potential eligible candidates

Overall, 95 patients were excluded due to IgG levels above the chosen threshold (without ongoing IgG-replacement therapy), 68 excluded for having detectable SARS-CoV-2-specific antibodies, 102 for presenting any type of autoimmune phenomena/disease or history of solid organ transplantation, 17 for history of neurologic problems and 59 for undergoing concomitant chemotherapy or targeted therapy for malignant diseases (Table 2).

**Table 2:** Reasons for exclusion of solicited and unsolicited applicants after questionnaire-driven contact.

Exclusion criteria	*N* (%)* Tot = 500
IgG-levels above the chosen threshold (changed prior to initiation of phase II)	95 (19)
Detectable SARS-CoV-2-specific antibodies	68 (13.6)
Auto-immune phenomena, disease or history of solid organ transplantation	102 (20.4)
Neurologic problems	17 (3.4)
Concomitant chemotherapy or targeted therapy	59 (11.8)
Withdrawal of interest for the study	100 (20)
Other (age-limit, inability to understand German, lack of inclusion criteria)	59 (11.8)

*Each pre-screening exclusion was assigned a single documented exclusion criterion; therefore, categories are mutually exclusive and percentages sum to 100%.

Moreover, we observed a high number of patients (*n* = 100) who ultimately declined their initial willingness to participate in the trial due to the burden associated with the study schedule, which required eight visits over a six-month period. Additional exclusion criteria (e.g., age-limit or inability to understand German), as well as the failure to confirm previously identified inclusion criteria led to the exclusion of the remaining candidates.

As far as the screening failures are concerned, patients were excluded for the following reasons that had not been detected during the first contact: SARS-CoV-2 antibody positivity (59.1%), autoimmune or immune-mediated conditions (15.9%; i.e., autoimmune gastritis, atopic eczema/neurodermatitis, lymphocytic granulomatosis associated with Common Variable Immunodeficiency, Evans Syndrome, Thrombocytopenia, and Psoriasis with Systemic Arthritis), other non-immune medical conditions (11.4%; i.e., elevated C-reactive protein, uncontrolled hypertension, previous stroke, and primary CNS lymphoma), elevated IgG levels (6.8%), low lymphocyte count (2.3%), disease progression (2.3%), and withdrawal of consent (2.3%).

### Duration of enrollment process and comparison with other trials of SARS-CoV-2 vaccines enrolling healthy volunteers

**Figure 2:**
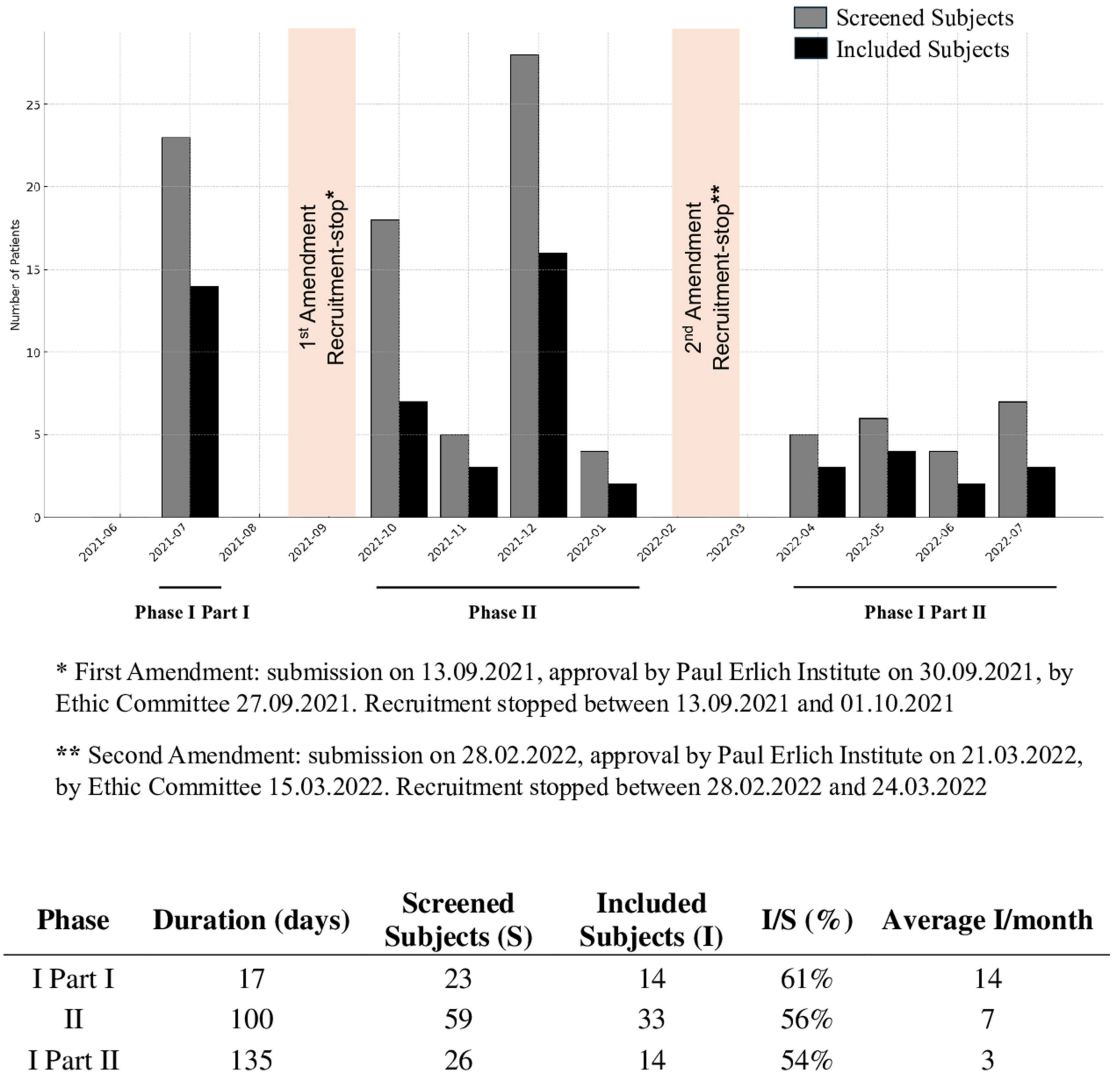
Screening and recruitment overtime.

Between July 6 and July 23, 2021, 14 patients were enrolled in Phase I Part I of the B-pVAC-SARS-CoV-2 clinical trial (i.e., 14 patients in 17 days). A further 33 patients were enrolled in Phase II between October 6, 2021, and January 13, 2022 (i.e., 33 patients in 100 days), while 14 new patients were enrolled in Phase I Part II of the trial between April 6 and August 18, 2022 (i.e., 14 patients in 135 days). The recruitment process of 68 trial participants in the three different centers took approximately 8 months, with an overall duration of the trial of approximately two and a half years, which is much longer than the recruiting and vaccination process of any other trial investigating novel vaccines against SARS-CoV-2 in healthy volunteers (9–16). Indeed, as reported in Table 3, the clinical development of the SARS-CoV-2 vaccines that are approved in western countries was achieved through clinical trials enrolling a high number of healthy donors, who were recruited and vaccinated within a few weeks (range of recruited/vaccinated volunteers per month: 30–12413 (9–16)). This contrasts with the recruitment rate of the B-pVAC-SARS-CoV-2 trial, which reached an average of eight patients per month, dropping to approximately 3 patients per month during the final Phase I Part II of the trial (Figure 2). Importantly, the prolonged trial duration primarily reflects challenges in identifying eligible participants rather than prolonged participation of individual subjects, representing not only a burden for the trial team, but leading to delayed results and impairing the possibility to extend the application of our product to additional patients, beyond the early phase trial.

**Table 3:** Duration trials investigating Vaxzevria/Covishield (AstraZeneca), Spikevax (Moderna), Comirnaty (Biontech), Jannsen (J&J) and CoVac1 (University of Tuebingen).

Phase	Number of patients	Duration (months)	Ratio patients/months
AstraZeneca*			
Phase I/II (9)	1,077	1	1,077
Phase III (10)	32,451	~4.5	7,211
Biontech*			
Phase I/II (11)	45	~1.5	30
Phase III/III (12)	43,448	~3.5	12,413
Moderna*			
Phase I (13)	45	~1	45
Phase III (14)	30,420	~3	10,140
J&J*			
Phase I/IIa (15)	805	~1	805
Phase III (16)	43,783	~4	10,946
CoVac-1			
Phase I (8)*	36	~2	18
Phase I/II (6)**	68	8	8.5

*Volunteers without relevant comorbidities.

**Patients with primary or acquired B-cell deficiencies.

### Trial specific amendments

The progress of the trial was further delayed by the submission of two trial amendments. These delays were partially anticipated, as preliminary safety and efficacy data, along with the assessments of the Data Safety and Monitoring Board (DSMB), had to be submitted to the German authorities before proceeding to the next trial phase. Moreover, the continuous exclusion of candidates who would have required an alternative effective immunization against SARS-CoV-2, prompted efforts to broaden eligibility through revisions of the approved criteria, a process carried out during the planned recruitment pause and amendment phase. To this end, data were retrieved from the literature or generated by the study team.

First, we showed that the IgG threshold initially introduced as inclusion criteria defining a relevant hypogammaglobulinemia and a very high likelihood of absent vaccine-induced humoral responses (i.e., IgG < 4 g/L) was too low. Indeed, emerging evidence demonstrated that patients with IgG levels between 4 and 5.5 g/L frequently remained unable to generate adequate SARS-CoV-2-specific antibody responses, supporting expansion of eligibility criteria (17). In addition, it was proved that hematologic patients undergoing treatment with Bruton-Tyrosine Kinase inhibitors (BTKi) (i.e., ibrutinib and acalabrutinib) or BCL-2 antagonist (i.e., venetoclax), who had been excluded from the trial, could profit from the investigational medical product (*in-vitro* data not shown), with no plausible safety issue. Overall, this allowed us to amend the IgG threshold (inclusion criteria edited) and to enroll subjects undergoing treatment with BTKi or BCL-2 antagonist (exclusion criteria removed). However, further questionable criteria, including (i) history of any neurological disorder, (ii) prophylactic treatment with monoclonal antibodies against SARS-CoV-2, (iii) low levels of SARS-CoV-2 specific IgG in patients regularly undergoing IgG-replacement therapy (collected during the pandemic) and (iv) autoimmune phenomena or previous transplantations, remained unchanged.

For each amendment, a recruitment stop was required. This, together with the time needed to generate and submit supporting evidence, resulted in an overall delay of approximately 3 months, negatively affecting the recruitment timeline.

## Discussion

Translational research represents a critical bridge between scientific discoveries and tangible medical solutions. However, a multitude of challenges arise when it comes to recruiting patients into early-phase clinical trials, as exemplified by the B-pVAC-SARS-CoV-2 trial. This Phase I/II study investigating CoVac-1, a peptide-based T-cell activator, aimed to address a critical medical need by evaluating CoVac-1 safety and efficacy in patients with acquired or inherited B-cell deficiencies (6). However, despite promising results of a Phase I trial with healthy volunteers (8) and a high patient demand, the recruitment process for the selected patient sub-population was particularly difficult, which is in line with previous reports highlighting that stringent criteria aimed at ensuring patients’ safety and trial’s validity often result in a limited pool of eligible candidates, with possible recruitment failure (18).

To understand the reasons for the slow enrollment, we kept track of exclusion criteria met by ineligible candidates, which helped to refine the protocol for subsequent parts of the trial. Particularly, we observed that the initially defined threshold of serum IgG < 4 g/L, which was introduced to limit the enrollment to patients with severe hypogammaglobulinemia that were for sure unable to mount an antibody response upon encountering the virus, was too low for successful recruitment. In fact, we screened patients without detectable antibodies after vaccination, who had higher IgG levels. As new reports confirmed that patients with IgG < 5.5 g/L were already at high risk of not developing SARS-CoV-2 antibodies (17), we could amend the clinical trial protocol, thus including single subjects who had been excluded before.

Similarly, patients undergoing treatment with BTKi or venetoclax had to be excluded from trial participation, due to unknown possible interactions with the study drugs. This led to significant limitations in terms of selecting potential study participants, as these agents are widely used in the treatment of hematological malignancies. To address this, we experimentally demonstrated that patients undergoing these treatments can develop T-cell mediated immune responses, which allowed us to delete this exclusion criteria.

Furthermore, the B-pVAC-SARS-CoV-2 trial faced difficulties in recruiting participants due to the concomitant dynamic of the pandemic. Indeed, beyond the complex patients’ profiles, the evolving landscape of therapeutic and prophylactic tools against SARS-CoV-2 had a significant impact on the recruitment process. With this regard, we observed that IgG cocktails used for IgG-replacement therapies improved throughout the pandemic and, in 2022, several patients who were regularly receiving IgG-replacement presented low detectable levels of antibodies against SARS-CoV-2. Similarly, with the introduction of SARS-CoV-2-specific monoclonal antibodies and their prophylactic use for patients undergoing B-cell depleting treatments, high levels of virus-specific antibodies could be detected in otherwise immunocompromised subjects. Since the presence of SARS-CoV-2-specific antibodies had been previously defined as an exclusion criterion and no differentiation between endogenous and exogenous antibodies was allowed, patients who received any type of passive immunization and were tested positive for SARS-CoV-2-specific antibodies had to be excluded from the trial, even in case of low antibodies-titers.

Furthermore, the incidence COVID-19 increased in the trial population, despite all isolation strategies. As any prior SARS-CoV-2 infection constituted an exclusion criterion, we were not allowed to enroll any patient with documented exposure to the virus, regardless of individual immune competence and the lack of specific humoral immunity. Moreover, subjects with any history of neurological problems, regardless of their non-immune origin (e.g., ischemic stroke) and low chances of recurrence, were excluded from the trial, due to precautionary safety considerations adopted during protocol development and concerns regarding attribution of potential neurological adverse events, which further shows how safety considerations need a more robust alignment with scientific and clinical evaluations of actual risks. This also supports the idea that it should be possible to tailor at least part of trial criteria to individual medical history, at discretion of the physicians and investigators leading the trial under the supervision of the DSMB.

Overall, this experience highlights the need to introduce some degree of flexibility in the selection of study participants, with (i) the foreseen possibility of adapting in/exclusion criteria, should no serious safety concerns emerge, and (ii) the opportunity of developing fast-tracks for an expedited editing (i.e., amendment) of an already approved protocol. The latter could be achieved through per-protocol established procedures that might involve only an expedited evaluation of protocol changes by the DSMB. This observation aligns with recent strategic perspectives in immuno-oncology, which emphasize the importance of adaptive and flexible trial designs to overcome rigid eligibility frameworks and accelerate recruitment without compromising safety (19).

We recognize that this standpoint could open to some ethical concerns, which are of great relevance in translational research, especially when it comes to investigating new treatment options in vulnerable patients, yet we repeatedly observed how the imperative of prioritizing safety, despite the pressing need for innovative therapies, can lead to stringent requirements to investigators, who are forced to edit parts of the research plan - as eligibility criteria or trial design - in order to obtain approval to conduct early phase trials (20, 21).

Practical challenges further compromise the success of early phase studies aimed at recruiting frail patients, who are already routinely undergoing treatments for their disease. Particularly, the burden placed on patients by the number of safety-visits can discourage participation, as observed during the B-pVAC-SARS-CoV-2 trial. This may inadvertently limit the group of willing candidates, potentially impacting the generalizability of trial results.

Additionally, the overarching framework of clinical trial recruitment itself presents obstacles. Traditional recruitment methods often rely heavily on clinicians’ referrals, which may not effectively reach all potential participants, particularly in selected patient populations. Effective outreach strategies that leverage digital platforms and communities are underutilized, which should be improved to support recruitment efforts (22–24). Here, the collaboration between researchers, clinicians, regulatory authorities, and patient advocacy groups becomes paramount (24). This can enhance patient identification by leveraging existing networks and knowledge, as well as by facilitating the dissemination of trial information and the development of flexible recruitment strategies (24, 25). Furthermore, broad communication of detailed inclusion and exclusion criteria may enhance recruitment efficiency by reducing inappropriate referrals, as we observed that many patients referred by colleagues met the main eligibility criteria but were subsequently excluded because of concomitant conditions that had not been recognized before referral.

Finally, although multicenter recruitment expands the available patient pool, our experience suggests that increasing the number of sites alone may not fully overcome barriers arising from restrictive eligibility criteria and rapidly evolving clinical contexts. For successful translational research, it is essential to develop new strategies based on the lessons learned from trials like the B-pVAC-SARS-CoV-2, in order to refine recruitment practices, adapting these to the evolving medical landscape, and ultimately bridging the gap between scientific innovation and patients’ needs. Our experience serves as a valuable example, highlighting both the potential of innovative therapies and the complexities of translating scientific discoveries into effective medical solutions for vulnerable populations. As we move forward, these insights should guide us, ensuring that groundbreaking therapies timely reach the patients who need them the most.

## Data Availability

The raw data supporting the conclusions of this article will be made available by the authors, without undue reservation.
